# Prevalence and molecular characterization of *Salmonella enterica* serovar Typhimurium from ice and beverages in Jakarta, Indonesia

**DOI:** 10.1186/s13104-019-4065-y

**Published:** 2019-01-21

**Authors:** Diana E. Waturangi, Eko Wiratama, Audora Sabatini

**Affiliations:** Faculty of Biotechnology, Atma Jaya Catholic University of Indonesia, Jalan Jenderal Sudirman 51, Jakarta, 12930 Indonesia

**Keywords:** *Salmonella enterica* serovar Typhimurium, Ice and beverages, Virulence, PCR

## Abstract

**Objective:**

The presence of *Salmonella enterica* serovar Typhimurium becomes a concern in relation to the safety of drinking water and ice. We detected and enumerated the bacteria from ice and beverages collected from several areas in Jakarta. Most Probable Number (MPN) and multiplex PCR method were used. Three sets of primers were used *rfbJ, fliC*, and *fljB*. Two Multiplex PCR’s were performed, the first is to detect the presence of *Salmonella* and the second is to confirm the positive isolate of *S. enterica* serovar Typhimurium.

**Results:**

A total of 50 beverages and 50 ices were collected MPN result ranged from < 3 to > 11,000 MPN/ml. The highest MPN value > 11,000 MPN/ml. The first Multiplex PCR result from beverages, 58% positively contained *Salmonella* spp. with amplification of *fliB* gene and no amplification of *rfbJ* and *fliC* genes. For ice samples, 2% positively contained *Salmonella* spp. with *rfbJ* gene amplification, 62% *fliB* gene and no amplification of *fliC* gene. The second Multiplex PCR results from beverages identified 21 positive isolates of *S. enterica* serovar Typhimurium. In which, 17 isolates contained *fljB* gene and 4 isolates contained both *fljB* and *rfbJ* genes. From ice, 17 isolates having both *rfbJ* and *fljB* genes.

## Introduction

*Salmonella* is the causative agent of salmonellosis. *Salmonella typhi* and *Salmonella paratyphi* are pathogenic exclusively for humans, causing systemic infections and typhoid fever, whereas *Salmonella enterica* serovar Typhimurium cause gastroenteritis [1].

The O factor of this bacteria determines the serogroup and the H factors define the serotype [2]. The flagella antigens H1 (phase 1) and H2 (phase 2) are encoded by *fliC* and *fljB* genes, respectively. These two genes are present at two different locations on the *Salmonella* chromosome. However, one of them is expressed at one time due to phase variation [3]. In *Salmonella*, the genes responsible for biosynthesis of the O antigens are normally grouped together on the chromosome in a gene cluster called *rfb* [4]. *Salmonella* comprise two species, which are *S. enterica* and *S. bongor* [5].

Salmonella infection is one of public health problems in developing countries, including Indonesia. Several publications reported high incidence of *Salmonella* infection in Indonesia [6].

Several techniques for the detection of *Salmonella* have been developed such as serology test, selective culture medium, and enzyme linked immunosorbent assay [7]. However, these methods have several limitations because of low sensitivity and specificity. The Polymerase Chain Reaction (PCR) is a rapid method for detection and identification of pathogens such as *Salmonella.* These molecular methods, such as multiplex PCR, are highly sensitive, very specific, fast and reproducible [8].

The aim of this study is to quantify the number of *Salmonella* contamination from ice and beverage samples using Most Probable Number (MPN) and Multiplex PCR method. The positive isolates were assayed for antibiotic resistance. In this study, we monitored and enumerated the presence of *Salmonella enterica* serovar Typhimurium, with particular reference to pathogenic potential and profiles for epidemiological analysis and risk assessment.

## Main text

### Methods

#### Samples collection

A total of 100 samples (50 ice and 50 beverages) were collected in Jakarta. The samples were collected from five different regions in Jakarta, designated as North, South, East, West, and Central Jakarta. In each case, 25 ml of sample was inoculated into 225 ml of Buffered Peptone Water (BPW) (Oxoid, Hampshire, England) [4].

All inoculated BPW were incubated at 37 °C for 16–20 h.

#### Detection and Enumeration of *S. enterica* serovar Typhimurium

Serial dilutions of 1:100 and 1:1000 Rappaport–Vassiliadis (RV) (MERCK, Darmstadt, Germany) were prepared in triplicate to pursue three-tube MPN-based enumeration [9]. All inoculated RV tubes were incubated at 42 °C for 24 h.

#### Genomic DNA extraction

As much as 1.5 ml of RV positive was centrifuged at 13,684×*g* for 3 min, then resuspended in 500 µl of sterile distilled water, boiled for 15 min, and used as cell lysates containing templates for PCR.

#### First multiplex PCR assays

PCR were done using primers *rfbJ, fliC,* and *fliB* [3]. We used *S. enterica* serovar Typhimurium S43 positive to *rfbJ and fljB* genes as positive control for multiplex PCR. A volume of 25 µl containing 12.5 µl of GoTaq (Promega, Madison, WI), 1 µl of each of the three primer-pairs (EUROGENTEC AIT, Singapore), 2.5 µl of DNA template, and 4 µl of nuclease free water (Promega, Madison, WI). PCR was programmed as follows: an initial denaturation at 95 °C for 3 min, followed by 20 cycles, each consisting of 95 °C for 1 min, 65 °C for 1 min, 72 °C for 30 s, and a final extension at 72 °C for 1 min.

#### Isolation of *S. enterica* Serovar Typhimurium and second multiplex PCR

To confirm the presence of *Salmonella*, positive RV broth culture were diluted from 10^0^ to 10^−4^ in 0.85% NaCl then spread onto Xylose Lysine Desoxycholate agar (XLD) (OXOID, Hamsphire, England) and incubated at 37 °C for 24 h.

The suspected *S. enterica* Serovar Typhimurium colonies were inoculated into Brain Heart Infusion Broth (BHIB) (Oxoid, Hampshire, England) and incubated at 37 °C, overnight. Eight or more suspected colonies were selected. 1.5 ml portions of turbid BHIB were centrifuged at 9503×*g* for 3 min, resuspended in 500 µl of sterile distilled water and boiled for 15 min. The supernatants were used as a template for the second multiplex PCR. The reaction and conditions of second multiplex PCR were similar to the first PCR.

#### Antibiotic susceptibility testing

The disc diffusion method (Kirby–Bauer) was used for antibiotic susceptibility test. The positive isolate of *S. enterica* serovar Typhimurium was cultured in BHIB medium and incubated at 37 °C for 18 h. The bacterial suspension was streaked in Mueller–Hinton agar (MH) (Benton-Dickinson, USA). A total of 9 antibiotics disc such as Streptomycin (10 µg), ampicillin (10 µg), trimetophrim (5 µg), ciprofloxacin (5 µg), erythromycin (15 µg), tetracycline (30 µg), kanamycin (30 µg), gentamicin (10 µg), and nalidixic acid (30 µg) (OXOID, Hampshire, England) were used. Zones of inhibition were measured and analyzed using CLSI standard [10].

### Results

Ice samples from North Jakarta, East Jakarta, Central Jakarta, and South Jakarta showed the same number of *Salmonella* spp, which was 2400 MPN/ml. Ice samples from West Jakarta showed a different number of *Salmonella* spp., which was > 11,000 MPN/ml (I.WJ.9 isolate). Most of the ice samples showed MPN value of < 30 MPN/ml.

Almost 34% of the samples (17 of 50) had MPN value of 230 MPN/ml. The highest MPN value > 11,000 MPN/ml was found in sample from East Jakarta (B.EJ.4). The lowest MPN value 3 MPN/ml was found in sample from Central Jakarta (B.CJ.1). The highest values from ice and beverage samples were the same (> 11,000 MPN/ml), but they were from different locations. Beverage sample was obtained from East Jakarta (B.EJ.4), while ice sample was obtained from West Jakarta (I.WJ.9).

Multiplex PCR results presented in Table 1 showed that from 50 beverage samples, there are 29 positive samples (58%), having positive result for *fljB* gene in all samples. The number of positive sample for each area is as follows: Central Jakarta 3 of 8 (37.50%), East Jakarta 9 of 9 (100%), West Jakarta 5 of 9 (55.55%), South Jakarta 7 of 16 (43.75%), and North Jakarta 5 of 8 (62.50%). The *rfbJ* and *fliC* genes were not found in all beverage samples (Table 1).

**Table 1:** Numbers and percentage of multiplex PCR of virulence genes from different region in Jakarta

Location	Beverages						Ice					
	Virulance gene						Virulance gene					
	*rfbJ* (663 bp)		*fliC* (183 bp)		*fljB* (526 bp)		*rfbJ* (663 bp)		*fliC* (183 bp)		*fljB* (526 bp)	
	No.	%	No.	%	No.	%	No.	%	No.	%	No.	%
Central Jakarta	(0/8)	0	(0/8)	0	(3/8)	37.5	(0/11)	0.00	(0/11)	0.00	(7/11)	63.63
East Jakarta	(0/9)	0	(0/9)	0	(9/9)	100	(1/7)	14.29	(0/7)	0.00	(7/7)	1.00
West Jakarta	(0/9)	0	(0/9)	0	(5/9)	55.55	(0/6)	0.00	(0/6)	0.00	(3/6)	0.50
South Jakarta	(0/16)	0	(0/16)	0	(7/16)	43.75	(0/15)	0.00	(0/15)	0.00	(8/15)	53.33
North Jakarta	(0/8)	0	(0/8)	0	(5/8)	62.5	(0/11)	0.00	(0/11)	0.00	(6/11)	54.55
Avegare	(0/50)	0	(0/50)	0	(29/50)	58	(1/50)	2	(0/50)	0	(31/50)	62

Thirty-one ice samples positively contained *Salmonella* spp. (62%) were found from the total of 50 ice samples. The number of positive sample for each area is as follows: Central Jakarta 7 of 11 (63.63%), East Jakarta 7 of 7 (100%), West Jakarta 3 of 6 (50%), South Jakarta 8 of 15 (53.33%), and North Jakarta 6 of 11 (54.55%). Most of them showed positive for *fljB* gene. One sample from East Jakarta (I.EJ.2) was found to carry two amplicons from *fljB* and *rfbJ* genes. The *fliC* gene was not found in all ice samples (Table 1).

From the 424 of suspected colonies, we found 21 positive isolates indicated as *S. enterica* serovar Typhimurium. Seventeen isolates (80.95%) had *fljB* gene and 4 isolates (19.05%) had both *fljB* and *rfbJ* genes. The *fliC* gene was not found. All of the positive isolates were found in samples collected from North, South, and East Jakarta.

**Fig. 1:**
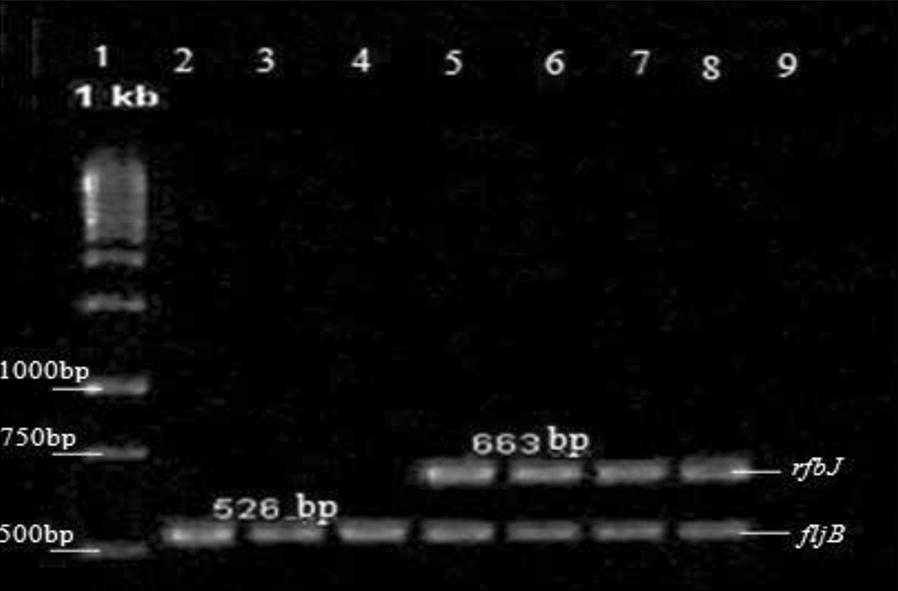
Result of Multiplex PCR of *rfbJ, fljB,* and *fliC genes* for identification of *S. enterica* serovar Typhimurium. Lane 1 shows the 1 kb DNA ladder, lane 2 to 4 show the PCR amplicons specific for *fljB gene* at 526 bp, lane 5 to 8 for *fljB* at 526 bp and *rfbJ* at 663 bp genes amplicon, lane 8 *S. enterica* serovar Typhimurium. S43 as positive control, and lane 9 for negative control

A total of 568 suspected colonies of *S. enterica* serovar Typhimurium were obtained from 31 ice positive samples. We found 17 positive isolates indicated as *S. enterica* serovar Typhimurium. One isolate (2.5%) was obtained from I.EJ.3 sample and 16 positive isolates (66.67%) were obtained from I.EJ.2 sample. They had *rfbJ* and *fljB* genes. All of these isolates were obtained from ice samples of East Jakarta (Table 2). The multiplex PCR products are shown in Fig. 1.

**Table 2:** Percentage and amplified product of *S. enterica* serovar Typhimurium isolate

Location	Beverages						Ice						
	Sample code	No. isolate	%	Virulance gene			Location	Sample code	No. isolate	%	Virulence gene		
				*rfbJ*	*fljB*	*fliC*					*rfbJ*	*fliC*	*fljB*
North Jakarta	B.NJ.2	(0/16)	0.00				North Jakarta	I.NJ.1	(0/8)	0.00			
	B.NJ.3	(0/15)	0.00					I.NJ.3	(0/4)	0.00			
	B.NJ.4	(0/8)	0.00					I.NJ.7	(0/20)	0.00			
	B.NJ.7	(1/16)	6.25		+			I.NJ.8	(0/2)	0.00			
	B.NJ.8	(0/8)	0.00					I.NJ.9	(0/4)	0.00			
South Jakarta	B.SJ.1	(0/8)	0.00					I.NJ.10	(0/8)	0.00			
	B.SJ.2	(0/24)	0.00					I.NJ.11	(0/15)	0.00			
	B.SJ.6	(0/8)	0.00				South Jakarta	I.SJ.3	(0/28)	0.00			
	B.SJ.7	(1/11)	9.09		+			I.SJ.4	(0/8)	0.00			
	B.SJ.8	(15/39)	38.46		+			I.SJ.5	(0/24)	0.00			
	B.SJ.13	(0/8)	0.00					I.SJ.6	(0/16)	0.00			
	B.SJ.6	(0/16)	0.00					I.SJ.7	(0/31)	0.00			
	B.SJ.13	(0/8)	0.00					I.SJ.8	(0/8)	0.00			
Central Jakarta	B.CJ.1	(0/16)	0.00					I.SJ.12	(0/8)	0.00			
	B.CJ.6	(0/16)	0.00					I.SJ.15	(0/8)	0.00			
	B.CJ.8	(0/24)	0.00				Central Jakarta	I.CJ.2	(0/8)	0.00			
East Jakarta	B.EJ.1	(2/24)	8.33	+	+			I.CJ.3	(0/24)	0.00			
	B.EJ.2	(0/8)	0.00					I.CJ.6	(0/30)	0.00			
	B.EJ.3	(0/8)	0.00				East Jakarta	I.EJ.1	(0/16)	0.00			
	B.EJ.4	(0/16)	0.00					I.EJ.2	(16/24)	66.67	+		+
	B.EJ.5	(2/24)	8.33	+	+			I.EJ.3	(1/40)	2.50	+		+
	B.EJ.6	(0/16)	0.00					I.EJ.4	(0/23)	0.00			
	B.EJ.7	(0/7)	0.00					I.EJ.5	(0/39)	0.00			
	B.EJ.8	(0/16)	0.00					I.EJ.6	(0/32)	0.00			
	B.EJ.9	(0/24)	0.00					I.EJ.7	(0/24)	0.00			
West Jakarta	B.WJ.6	(0/16)	0.00				West Jakarta	I.WJ.1	(0/20)	0.00			
	B.WJ.7	(0/8)	0.00					I.WJ.2	(0/16)	0.00			
	B.WJ.8	(0/8)	0.00					I.WJ.7	(0/16)	0.00			
	B.WJ.9	(0/8)	0.00					I.WJ.8	(0/32)	0.00			
								I.WJ.9	(0/8)	0.00			
								I.WJ.11	(0/24)	0.00

The antibiotic resistant patterns of 21 isolates from beverage and 17 isolates showed that for beverages 66.67% resistance for streptomycin (10 µg), 66.67% for ampicillin (10 µg), 9.57% for trimethoprim (5 µg), 28.57% for ciprofloxacin (5 µg), 66.67% for erythromycin (15 µg), 52.38% for tetracyclin (30 µg), 28.57% for kanamycin (30 µg), 71.42% for gentamicin (10 µg), and 28.57% for nalidix acid (30 µg). The percentages of resistance in ice isolates are 82.35%; 23.53%; 0%; 17.65%; 82.35%; 23.53%; 23.53%; 76.47%, and 17.65% respectively. The highest percentage of resistance was shown by streptomycin (10 µg) and erythromycin (15 µg).

### Discussion

The MPN value indicates the risk of transmitting diseases to humans through beverages and ices. Several publications have reported specific detection of *S. enterica* serovar Typhimurium by multiplex PCR using *rfbJ, fliC*, and *fljB* genes [8]. The flagella antigens H1 (phase-1) and H2 (phase-2) are encoded by *fliC* and *fljB* genes, respectively [11].

From the first multiplex PCR, 29 samples positive from beverages, and 31 samples positive from ice. Both samples was found to be positive *fljB* gene, except ice sample from East Jakarta (I.EJ.2) which gave positive amplification for both *fljB* and *rfbJ* genes. There were no positive amplification of *fliC* gene from both ice and beverage samples.

From the second multiplex PCR, we found 17 positive isolates of *S. enterica* Serovar Typhimurium from ice sample obtained from East Jakarta. One isolate (2.5%) was obtained from I.EJ.3 sample and 16 isolates (66.67%) were obtained from I.EJ.2 sample. These isolates gave positive amplification of both *rfbJ* and *fljB* genes. Therefore, the contamination might occur from the same source of contamination, such as source of water. The environment near the location in which the samples were collected were unhygienic that might contribute to the *S. enterica* serovar Typhimurium contamination.

The second multiplex PCR result from beverages showed 21 positive isolates shows of *S. enterica* serovar Typhimurium, the samples were obtained from North, South, and East Jakarta. *S. enterica* serovar Typhimurium contamination was more distributed in beverage samples than in the ice samples. We found more *S. enterica* serovar Typhimurium isolated from beverages than ice. It indicates that the beverage samples were more contaminated by *S. enterica* serovar Typhimurium than ice sample, because the source of contamination can come from raw water usage for beverages or due to unhygienic storage.

In this study, we obtained *rfbJ* and *fljB* genes from *S. enterica* serovar Typhimurium isolates (lack of *fliC* amplicon). Lim et al. [12] reported that three sets of primer targeted *rfbJ, fljB,* and *fliC* genes caused specific identification of O4, H:1,2 and H:i antigenic properties, respectively. Only *S. enterica* serovar Typhimurium had the antigenic structure combination of O4, H:1,2, and H:i out of about 2000 serovars. Besides that, other studies reported that PCR produced 663, 526, 284, and 183 bp amplicons from *rfbJ, fljB, invA,* and *fliC*, respectively, in all 9 serovars [13].

Shanmugasundaram et al. [14] reported that isolates which showed positive for *rfbJ* gene were confirmed as *S. enterica* serovar Typhimurium. It could identified two of seven biochemically suspected *Salmonella* isolates that were later confirmed as *S. enterica* serovar Typhimurium. The *fliC* amplification showed a large number of serovars of *S. Enterica* group, including *S. enterica* serovar Typhimurium. The *fljB* gene was detected in all strains of *S. enterica* with the exception of *S. enterica* serovar Typhi.

In this study, we obtained *Salmonella enterica* serovar Typhimurium that lack of phase-1 antigen (*fliC* gene). Monophasic variants were different from monophasic serovars because monophasic serovars did not have the second phase flagellar antigen [15]. We conclude that *Salmonella enterica* serovar Typhimurium isolates that we obtained in this study were monophasic variant.

Antibiotic-resistant *Salmonella* isolates are important for public health. All of the *S. enterica* serovar Typhimurium isolates we identified were resistant to at least 2 antibiotics. The widespread use of antibiotics as a treatment for diseases and to promote growth in the livestock and poultry industries has resulted in the emergence of resistant strains [13].

*Salmonella enterica* serovar Typhimurium phage type DT104 strains, which contains an antibiotic gene cluster resistance to ampicillin, chloramphenicol/florfenicol, streptomycin/spectinomycin, sulphonamides, and tetracycline [16, 17]. *S. enterica* serovar Typhimurium resistant to gentamicin and kanamycin [18].

This study has showed various results of antibiotic resistance in *S. enterica* serovar Typhimurium isolates from ice and beverage samples. We found high incidence of *Salmonella* spp. which was resistant to multiple antibiotics. The improper use of antibiotics to treat pathogenic bacteria associated with diarrheal disease in Indonesia might increase antibiotic resistance.

## Limitation

Multiplex PCR might detect other Salmonella which also have similar virulence genes.
