# Correction to: Prevalence and molecular characterization of *Salmonella enterica* serovar Typhimurium from ice and beverages in Jakarta, Indonesia

**DOI:** 10.1186/s13104-019-4131-5

**Published:** 2019-02-19

**Authors:** Diana E. Waturangi, Eko Wiratama, Audora Sabatini Theresia

**Affiliations:** Faculty of Biotechnology, Atma Jaya Catholic University of Indonesia, Jalan Jenderal Sudirman 51, Jakarta, 12930 Indonesia

## Correction to: BMC Res Notes (2019) 12:45 10.1186/s13104-019-4065-y

Following publication of the original article [1], the authors reported that the family name of one of the authors was omitted. In this Correction the incorrect and correct author name are shown. The original publication of this article has been corrected.

Originally the author name was published as:

- Audora Sabatini

The correct author name is:

- Audora Sabatini Theresia
